# Selective Enzymatic Transformation to Aldehydes *in vivo* by Fungal Carboxylate Reductase from *Neurospora crassa*

**DOI:** 10.1002/adsc.201600914

**Published:** 2016-10-04

**Authors:** Daniel Schwendenwein, Giuseppe Fiume, Hansjörg Weber, Florian Rudroff, Margit Winkler

**Affiliations:** ^1^ acib GmbHPetersgasse 148010GrazAustria; ^2^ Institute of Molecular BiotechnologyGraz University of TechnologyNAWI GrazPetersgasse 148010GrazAustria; ^3^ Institute of Organic ChemistryGraz University of TechnologyNAWI GrazStremayrgasse 98010GrazAustria; ^4^ Institute of Applied Synthetic ChemistryTU WienGetreidemarkt 9/OC−1631060ViennaAustria

**Keywords:** aldehydes, biocatalysis, carboxylate reductase, carboxylic acids, flavours and fragrances

## Abstract

The enzymatic reduction of carboxylic acids is in its infancy with only a handful of biocatalysts available to this end. We have increased the spectrum of carboxylate‐reducing enzymes (CARs) with the sequence of a fungal CAR from *Neurospora crassa* OR74A (*Nc*CAR). *Nc*CAR was efficiently expressed in *E. coli* using an autoinduction protocol at low temperature. It was purified and characterized *in vitro*, revealing a broad substrate acceptance, a pH optimum at pH 5.5–6.0, a *T*_m_ of 45 °C and inhibition by the co‐product pyrophosphate which can be alleviated by the addition of pyrophosphatase. The synthetic utility of *Nc*CAR was demonstrated in a whole‐cell biotransformation using the *Escherichia coli* K‐12 MG1655 RARE strain in order to suppress overreduction to undesired alcohol. The fragrance compound piperonal was prepared from piperonylic acid (30 mM) on gram scale in 92 % isolated yield in >98% purity. This corresponds to a productivity of 1.5 g/L/h.

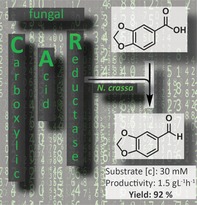

## Introduction

**Scheme 1:**
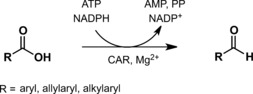
Reduction of carboxylic acids to the respective aldehydes by *Neurospora crassa* SY7A CAR.

Carboxylic acids show little reactivity and require a high level of activation to participate in chemical reactions. Particularly their direct reduction is a strongly endergonic process. A mild and selective alternative to chemical reduction protocols^[1–3]^ is the enzymatic reduction of carboxylic acids. Carboxylate reductase (CAR) enzymes exhibit a broad substrate tolerance for the conversion of organic acids to the respective aldehydes.^4^ However, only few CAR enzyme sequences have been elucidated^5^, ^6^, ^7^, ^8^, ^9^, ^10^, ^11^, ^12^ and are available for biocatalysis to date, although Nature provides a great versatility of organisms with the capability to catalyze this reaction.^[4,13–18]^ The reduction of salicylic acid,^19^ benzoic acid and derivatives^20^ as well as cinnamic acid and derivatives^21^ has been observed in the ascomycete *Neurospora crassa* (*Nc*). The enzyme that was able to reduce aromatic carboxylates has been isolated from the fungus and characterized.^20^,^22^ The classification of this enzyme as aryl‐aldehyde:NADP oxidoreductase^21^ was based on its substrate scope which appeared to be restricted to compounds with aromatic ring structures. Aliphatic acids and amino acids were not reduced by this enzyme.^23^ The carboxylate reduction is dependent on ATP, NADPH and magnesium ions (Scheme 1), leading eventually to its classification as an E.C. 1.2.1.30 enzyme. These E.C. 1.2.1.30 type CARs are comprised of three domains: an adenylation domain (A‐domain), a phosphopantetheinyl binding domain, and a reductase domain (R‐domain). They require post‐translational modification of the respective domain by phosphopantetheinylation.^24^

Aldehydes such as vanillin and hexanal account for flavour and fragrance sensing and are commercially used in the food and perfumery industry. Typically, aldehydes are reactive moieties and can undergo many chemical reactions such as reductive amination, aldol reactions, reduction to alcohol, acetal formation, Grignard reaction and many others. Therefore, they are widely employed as building blocks and used as precursors to pharmaceuticals and agrochemicals. Piperonal (Heliotropin) is an aromatic aldehyde used in many fragrances and also as a flavour component.^25^ It can be found in some essential oils, vanilla and camphor,^25^, ^26^, ^27^ and has been shown to have antibacterial^28^ and anxiolytic effects.^29^ Moreover, some piperonal derivatives show a potential anticancer effect,^30^,^31^ antileishmanial activity,^32^ and other pharmacological effects.^33^

## Results and Discussion

**Figure 1:**
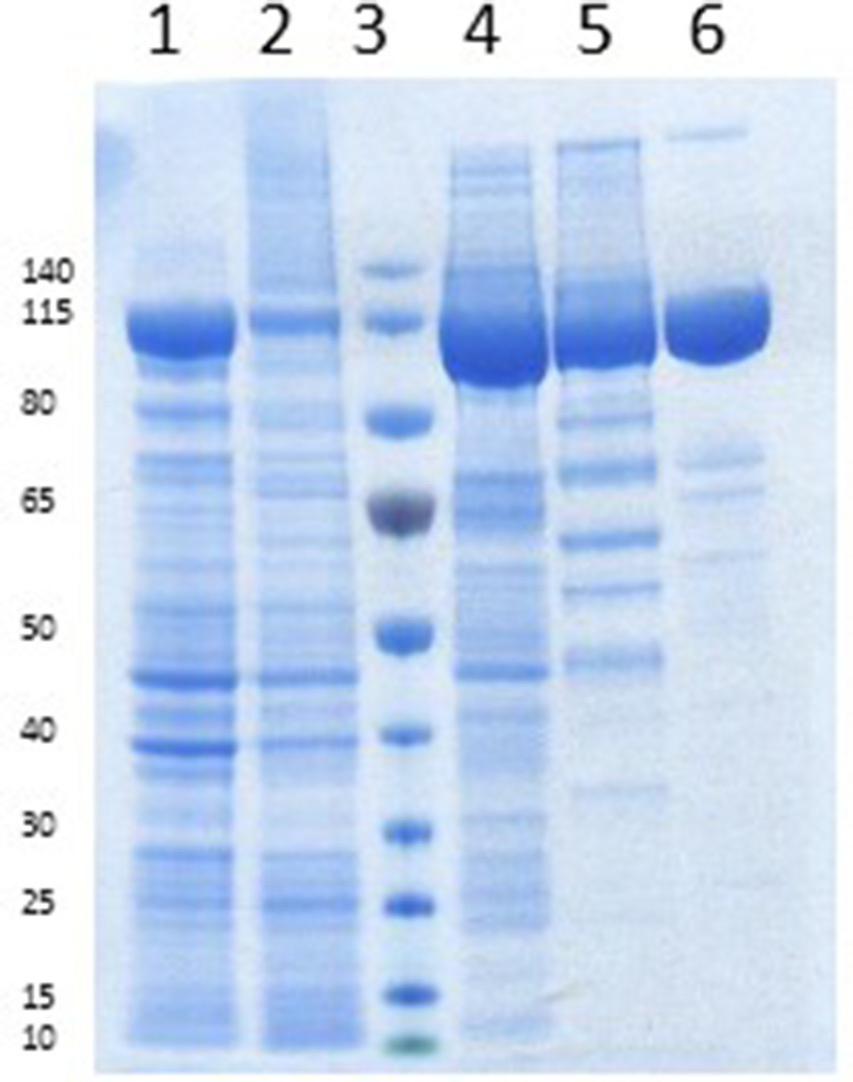
SDS 4–12% Bis‐Tris‐gel of *Nc*CAR preparations (1 h at 200 V in MOPS‐buffer; staining: SimplyBlue^TM^ SafeStain solution). Lane 1: cell free extract. Lane 2: cell pellet dissolved in 6 M urea. Lane 3: PageRuler^TM^ Prestained Protein Ladder (10–140 kDa). Lane 4: *Nc*CAR after Ni‐affinity chromatography. Lane 5: *Nc*CAR after removal of the His‐Tag with TEV‐protease and Ni‐affinity chromatography. Lane 6: *Nc*CAR from lane 4 after gel filtration.

With the aim to identify the primary sequence of *Neurospora crassa* CAR, four literature known CAR enzyme sequences (*Ni*CAR:^5^ Q6RKB1.1, *Mm*CAR:^6^ B2HN69, *Sr*CAR:^8^ WP_013138593.1 and *At*CAR:^7^ XP_001212808.1) were used as templates for a blastp search against the Gen‐Bank non‐redundant protein sequences of *Neurospora crassa* (taxid:5241). The hit with highest scores was a hypothetical protein with NCBI accession code XP_955820.1. On the sequence level, this protein showed low identities with the original search templates *Ni*CAR, *Mm*CAR, *Sr*CAR and *At*CAR (17.3%, 17.5%, 18.5% and 22.5%, respectively), and also low identities with CARs that have been published during this study: *Mn*CAR,^9^ *Tv*CAR,^10^ *Ms*CAR,^11^ *Nb*CAR^11^ and *Sb*CAR^12^ (16.4%, 26.1%, 17.7%, 17.3% and 22.4%, respectively). A codon optimized synthetic gene coding for XP_955820.1 from *N. crassa* OR74A was expressed with an *N*‐terminal TEV cleavable HIS‐tag from the multiple cloning site 2 of the pETDUET1 vector. For the essential post‐translational modification of CAR enzymes,^24^ *E. coli* phosphopantetheinyl transferase (NCBI accession code CAQ31055.1) was simultaneously expressed from multiple cloning site 1.^10^ *E. coli* BL21 (DE3) Star served as the host and the cells were cultivated under autoinduction conditions.^34^ The expected 120 kDa protein was termed *Nc*CAR and purified *via* Ni‐affinity chromatography and gel‐filtration (Figure 1). Notably, *Nc*CAR was expressed very efficiently in *E. coli* as a soluble protein, in contrast to another fungal CAR from *Trametes versicolor* under identical conditions.^10^ Other published fungal CARs ATEG03630 (*An*CAR) and StbB (*Sb*CAR) have been heterologously expressed in *S. cerevisiae* 7 and *Aspergillus oryzae*,^12^ respectively.

To confirm that XP_955820.1 codes for a carboxylate reductase, a spectrophotometric NADPH depletion assay was applied to screen for activity towards the reduction of a panel of carboxylic acids (Table 1). For certain substrates, however, the spectrophotometric assay was not suitable due to absorption of the acid or the aldehyde at the detection wavelength or due to their low solubility in aqueous system. Vanillic acid, e.g., is reduced to vanillin by *Nc*CAR, as determined by HPLC analyses.

**Table 1:** Selected results of spectrophotometric screening for substrates of *Nc*CAR.

Substrate	Specific activity [U/mg]^[a]^
benzoic acid^[b]^	2.74±1.17
2‐hydroxybenzoic acid^[c]^	0.49±0.17
2‐methoxybenzoic acid^[c]^	0.74±0.29
3‐hydroxybenzoic acid^[c]^	1.00±0.24
3‐methoxybenzoic acid^[c]^	1.71±0.64
4‐hydroxybenzoic acid^[c]^	0.68±0.55
4‐methoxybenzoic acid^[c]^	2.85±1.22
benzyloxyacetic acid^[b]^	0.52±0.03
3‐phenylpropionic acid^[c]^	0.69±0.29
pyridine‐2‐carboxylic acid^[b]^	1.55±0.18
**1a** cinnamic acid^[b]^	2.04±0.81
**2a** piperonylic acid^[c]^	2.22±0.85

^[a]^ One activity unit is defined as the amount of enzyme preparation catalyzing the oxidation of 1 μmol NADPH per minute. ^[b]^ Substrate dissolved in 0.1 M KOH. ^[c]^ Substrate dissolved in DMSO.

The spectrophotometric assay was also used to determine the biochemical characteristics of *Nc*CAR *in vitro*. The highest initial rate activity was observed at 55 °C, however, stability measurements indicated complete loss of activity upon incubation of the enzyme at this temperature for 90 min, whereas the residual activity after incubation at 35 °C was 84±17%. This is consistent with the *T*_m_ of *Nc*CAR of 45.3 °C.

**Figure 2:**
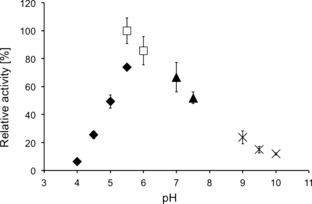
Relative activities of *Nc*CAR at different pH values. ⧫: citrate buffer, □: MES buffer, ▴: Tris‐HCl buffer, **×**: glycine buffer.

The published pH optimum of the enzyme purified from *N. crassa* was pH 7.7–8.1 as determined using endpoint quantification of radiolabelled salicylic aldehyde formation.^20^ The pH optimum of our recombinant *Nc*CAR appeared to be at pH 5.5–6.0 using the above mentioned spectrophotometric initial rate measurement with cinnamic acid (**1a**) as the substrate (Figure 2). This result was confirmed by an endpoint measurement of cinnamaldehyde (**1b**) formation by HPLC (data not shown). These diverging results may partly be a consequence of unequal reaction conditions, additional post‐translational modifications of the native *Neurospora crassa* CAR or due to differences in the amino acid sequence as a result of different strain backgrounds (SY7A *versus* OR74A).

Apparent kinetic parameters were determined with *Nc*CAR after cleavage of the HIS‐tag, using the spectrophotometric assay. The K_m_ value for **1a** at 28 °C was 445±50 μM. The turnover number k_cat_ was 4.57 s^−1^ and v_max_ 2.33±0.06 μmol min^−1^ mg^−1^. The K_m_ value for **2a** at 28 °C was 173±22 μM. The turnover number k_cat_ was 7.46 s^−1^ and v_max_ 3.70±0.12 μmol min^−1^ mg^−1^.

*In vitro* biotransformations coupled to HPLC analyses showed that carboxylic acids were often not converted quantitatively by *Nc*CAR. Assuming a limitation by ATP degradation, we performed ^1^H NMR in combination with time‐resolved ^31^P NMR analysis. ^31^P NMR had previously been used to aid the identification of the acyl‐adenylate intermediate in *Ni*CAR‐mediated bioreduction^[35]^ but not to monitor the reaction or to investigate the nature of the phosphorus‐containing species.^36^ First experiments towards the elucidation of the co‐products from ATP in *N. crassa* CAR reductions indicated the formation of ADP and phosphate,^21^,^37^ whereas later studies by the same author(s) showed that AMP and pyrophosphate were formed.^22^,^37^ Figure 6 shows ^31^P NMR signals of the reference measurements (*top*) and different time points of the reaction in the presence of *Nc*CAR. As expected, **1a** reduction resulted in AMP, pyrophosphate and NADP^+^. Whereas the signals of ATP and NADPH decreased, AMP and pyrophosphate were immediately formed after starting the reaction, however, the reaction did not continue beyond a certain point when a white precipitate emerged, although **1a**, NADPH and ATP were still present according to ^1^H and ^13^P NMR, respectively (Supporting Information, Figure S5). Denaturation of the *Nc*CAR was ruled out because it was stable in separate experiments under identical conditions. Controls with all potential by‐products showed that the precipitate was pyrophosphate. Consistent with the observations from the Prather group with *Ni*CAR,^11^ these results indicate the inhibition of *Nc*CAR by the co‐product pyrophosphate. An experiment including pyrophosphatase showed that the concentration of **1b** can indeed be increased‐two fold *in vitro* (Supporting Information, Figured S4).

**Figure 3:**
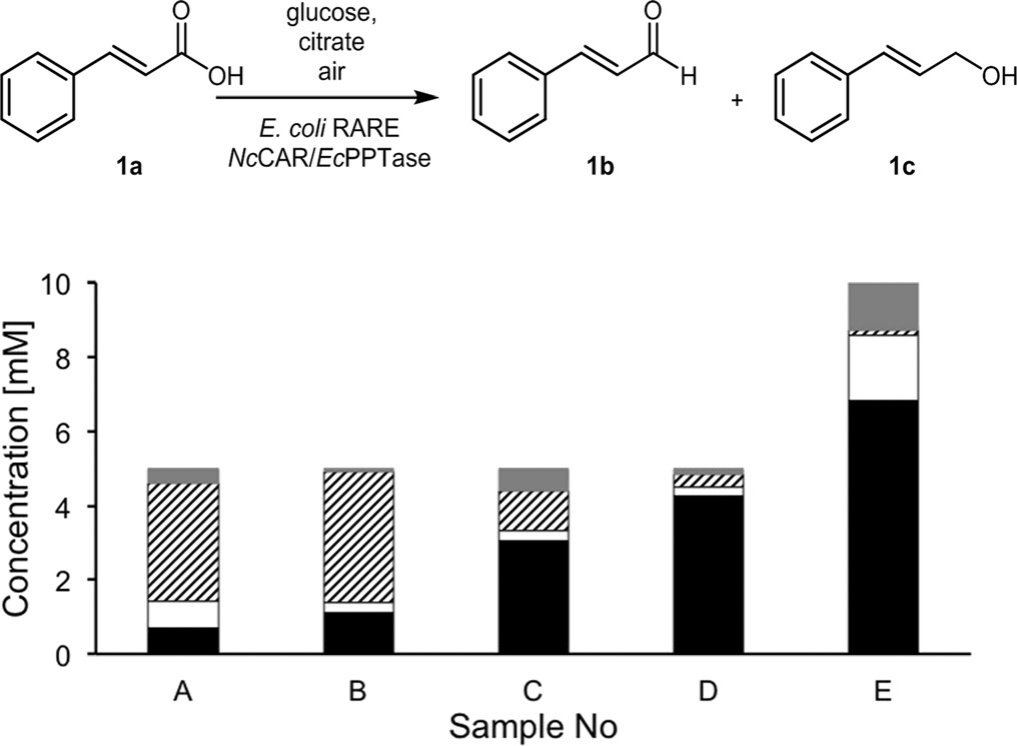
Optimization of whole cell biotransformation conditions. black: **1b**; striped: **1a**; white: **1c**; grey: not recovered. The standard conditions were 20 mg wet cells in 1 mL final volume, supplemented with 60 mM glucose, 10 mM sodium citrate, 2 mM Mg^2+^ and 5 mM **1a** in 15‐mL Pyrex tubes, which were incubated for 3 h. These standard conditions were modified as follows A: 70 mM sodium citrate, incubation time 20 h; B: 70 mM sodium citrate; C: 20% of *n*‐hexane; D: 50% of *n*‐hexane; E: 50% *n*‐hexane, 10 mM **1a**, 60 mg wet cells.

Currently, the most economical way of utilizing CAR enzymes on a preparative scale is their use *via* whole cell biocatalysts that allows cost effective regeneration of the required co‐factors (ATP, NADPH).^8^,^38^, ^39^, ^40^ At the same time, the enzymatic background of bacterial cells rapidly reduces aldehydes (**b**) to the corresponding alcohols (**c**) which minimizes the oxidative and electrophilic stress responses induced by the reactive carbonyl group in aldehydes.^[39,41]^ To circumvent **b** reduction, we opted for *E. coli* K‐12 MG1655 RARE^42^ – a knock‐out strain with reduced aromatic aldehyde reduction – as a host for whole cell mediated aldehyde preparation. Despite this deliberate choice, initial experiments towards the reduction of the model substrate cinnamic acid **1a** to **1b** showed significant amounts of the undesired alcohol **1c**. First whole cell biotransformations yielded only 14% of **1b** after 20 h and also 14% of **1c**. Reduction of the incubation time and co‐factor recycling components improved the formation of **1b** and decreased the amounts of **1c**. Further increase of **1b** was achieved by using hexane as a second phase, in order to circumvent product inhibition or toxicity, to finally 6.8 mM of **1b** (Figure 3).

**Figure 4:**
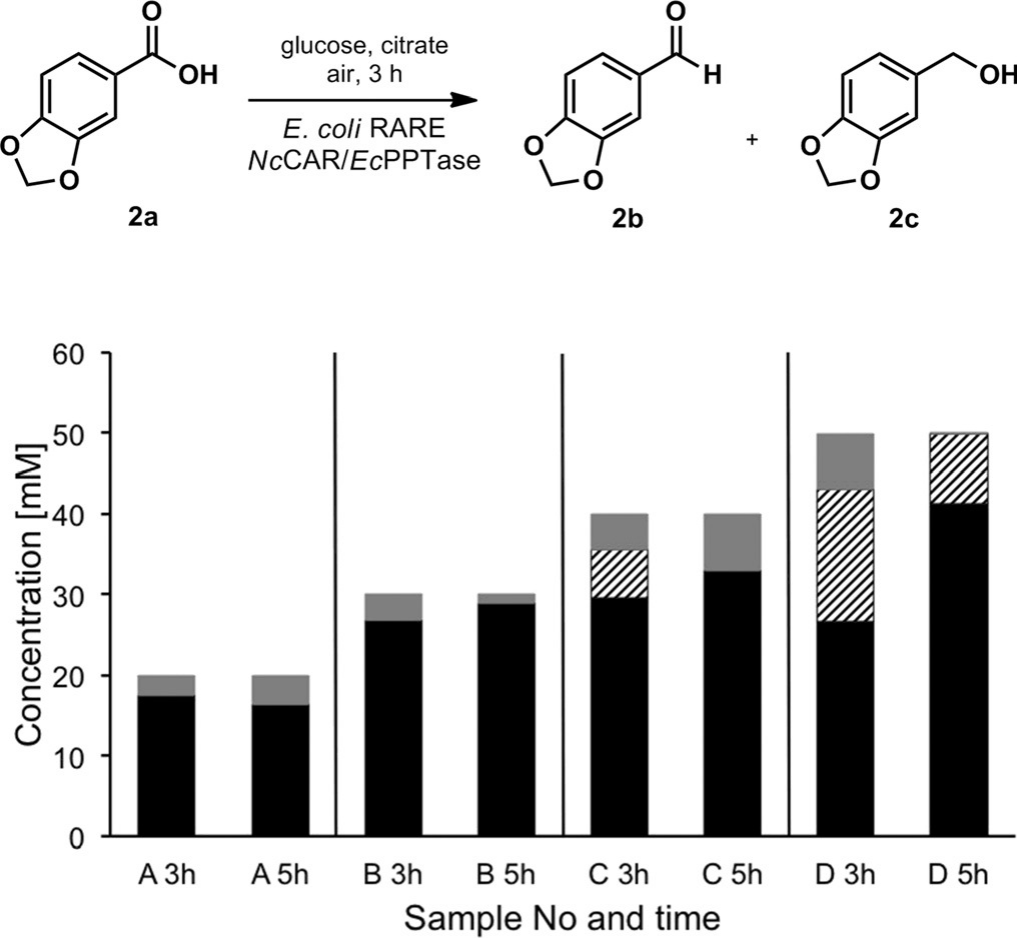
Whole cell biotransformation of increasing amounts of **2a** with *Nc*CAR expressed in *E. coli* K‐12 MG1655 RARE. black: **2b**; striped: **2a**; white: **2c**; grey: not recovered. The standard conditions were 500 mg wet cells in 1 mL final volume, supplemented with 120 mM glucose, 20 mM sodium citrate and 4 mM Mg^2+^ in 100 mL baffled flasks. A: 20 mM **2a**; B: 30 mM **2a**; C: 40 mM **2a**; D: 50 mM **2a**.

**Figure 5:**
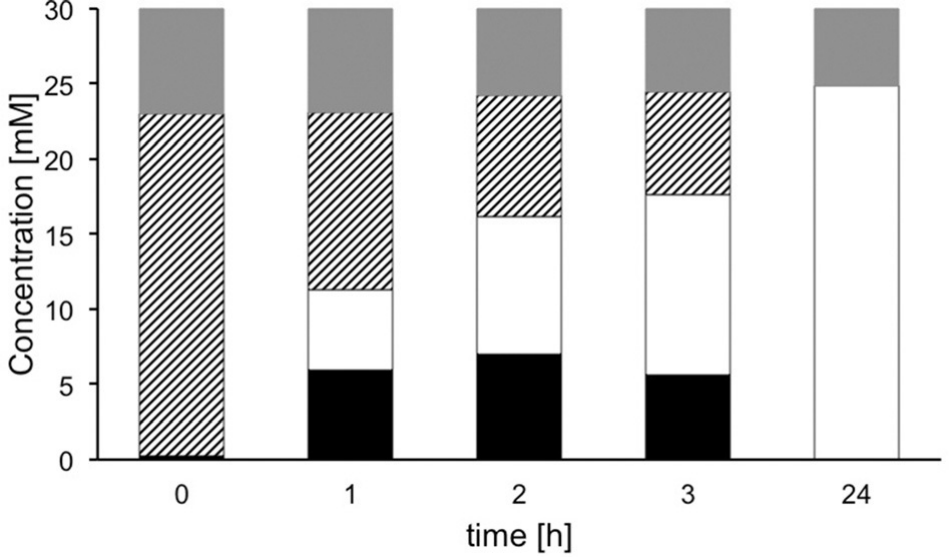
Time course of whole cell biotransformation of 30 mM **2a** using *Nc*CAR expressed in *E. coli* BL21 (DE3) Star. black: **2b**; striped: **2a**; white: **2c**; grey: not recovered. For the reaction scheme, see Figure 4.

**Scheme 2:**
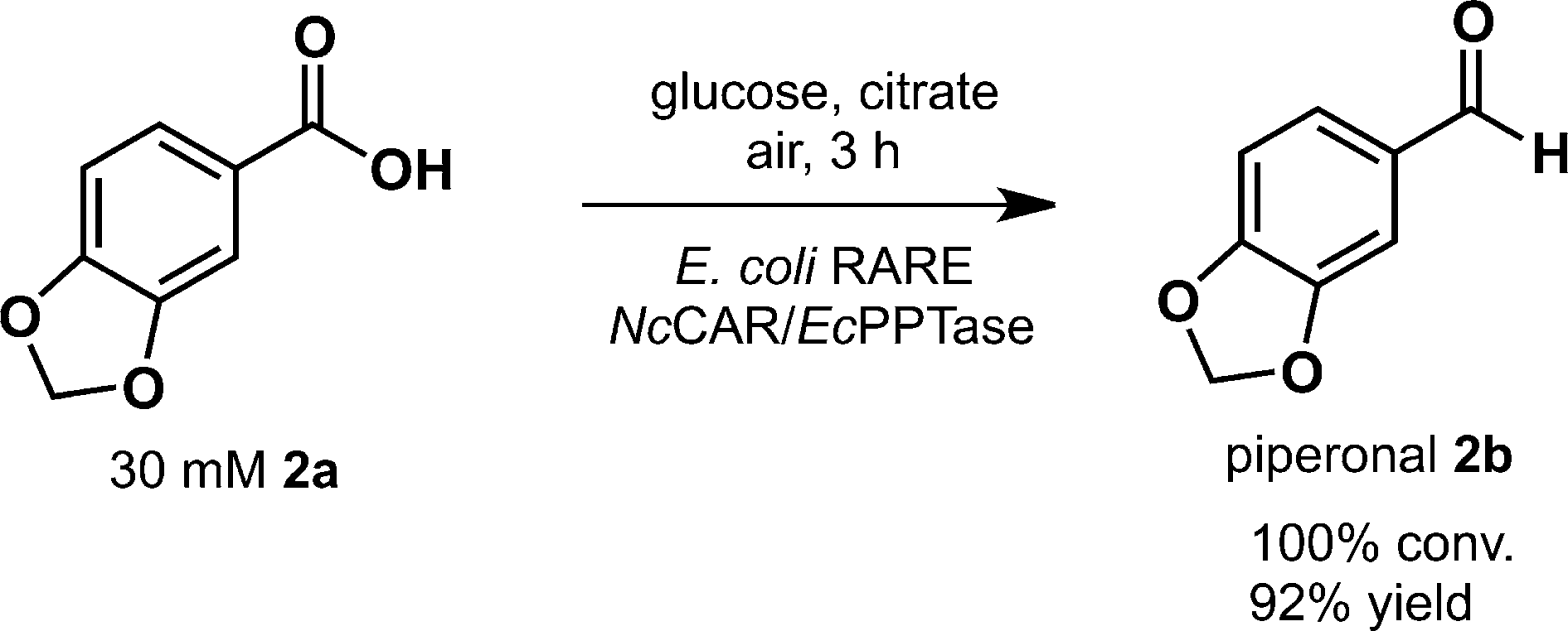
Preparative scale reduction of **2a** to piperonal **2b** by *Nc*CAR expressed in engineered *E. coli*.

Our goal was to demonstrate the synthetic utility of *Nc*CAR for the selective preparation of valuable aldehydes such as piperonal (**2b**).When we applied conditions B (Figure 3) to the preparation of **2b** using *Nc*CAR expressed in *E. coli* RARE, we were delighted to find that formation of **2c** was completely suppressed and **2b** was produced very efficiently even without the need for *n*‐hexane to increase product titers. Within 3 h, 30 mM of **2a** were fully converted to **2b** (Figure 4, B 3 h) and 40 mM, corresponding to 6.6 g L^−1^ were quantitatively reduced within 5 h (Figure 4, C 5 h). Further increase of the substrate concentration to 50 mM led to full conversion in less than 24 h and the maximal observed concentration of **2b** was 49.8 mM (data not shown), corresponding to 7.5 g L^−1^. In comparison, *E. coli* BL21 (DE3) Star expressing *Nc*CAR used under conditions identical to those described in Figure 4, B, gave predominantly the alcohol **2c** (Figure 5). Finally, piperonal **2b** was synthesized as follows: 1.99 g (30 mM) of **2a** was quantitatively reduced in two parallel batches exclusively to **2b** within 3 h (Scheme 2).

The product was extracted into *n*‐hexane and crystallized in >98% purity to give 1.66 g of **2b** in 92% isolated yield. This corresponds to an excellent productivity for enzymatic carboxylate reductions of >1.5 g L^−1^ h^−1^. The only report of comparable productivity concerned the *Nocardia iowensis* CAR‐mediated preparation of vanillin after extensive optimization. Despite co‐expression of GDH and the addition of XAD‐2 resin as a product scavenger, the mass balance did not attain 100% and vanillyl alcohol formation reduced the vanillin yield.^43^ The co‐expression of GDH may also improve piperonal productivity.

## Conclusions

The family of E.C.1.2.1.30 carboxylate reductase enzymes (CARs) was extended by a distinct enzyme sequence from *Neurospora crassa* OR74A (*Nc*CAR). Except for its optimal pH, *Nc*CAR seems to resemble aryl‐aldehyde:NADP oxidoreductase which was purified from *Neurospora crassa* SY7A in the 1970s. *Nc*CAR is well expressed in *E. coli*, which represents an excellent basis for biocatalytic applications. Herein we have designed a whole cell biocatalyst for the selective preparation of aldehydes and investigated the critical parameters for its use, such as the cell density, nature and amounts of additives, and substrate concentrations. *Nc*CAR was most efficiently used for the selective preparation of the flavour/fragrance aldehyde piperonal, reaching a productivity of approximately 1.5 g L^−1^ h^−1^ of the desired product without residual piperonylic acid or contamination by over‐reduction product.

## Experimental Section

### General

ATP was obtained from Roche Diagnostics. NADPH and MES were purchased from Roth, IPTG from Serva, and MgCl_2_ from Merck. HPLC‐MS grade acetonitrile was purchased from J.T.Baker/Avantor Performance Materials, Deventer, The Netherlands. All other chemicals were obtained from Sigma–Aldrich/Fluka or Roth and used without further purification.

*E. coli* cells were cultivated in an RS 306 shaker (Infors, Bottmingen, Switzerland) and Multitron shakers (Infors AG), and the cells were harvested with an Avanti J‐20 centrifuge (Beckman Coulter). Cell pellets were disrupted by a 102C converter with a Sonifier 250 (Branson, Danbury, CT), and the cell‐free extract was obtained by centrifugation in an Ultracentrifuge Optima LE80K (Beckman). Enzymes were purified in an ÄKTAPure 100 with a fraction collector F9‐C (Unicorn 6.3 software; GE Healthcare) and desalted with ÄKTAPrime (PrimeView 5.0 software; GE Healthcare) or alternatively purified using a gravity flow protocol. The protein‐containing fractions were pooled, the buffer exchanged for 50 mM MES buffer, pH 7.5, containing 10 mM MgCl_2_, 1 mM EDTA, and 1 mM DTT and aliquots of the resultant protein solution were shock frozen in liquid nitrogen and stored at −80 °C. Protein concentrations were determined with a Nanodrop 2000c spectrophotometer (Thermo Scientific). Reactions were performed on a Thermomixer comfort (Eppendorf). HPLC/MS analysis was carried out on an Agilent Technologies 1200 Series equipped with G1379B degasser, G1312B binary pump SL, G1367C HiP‐ALS SL autosampler, a G1314C VWD SL UV detector, G1316B TCC SL column oven and a G1956B MSD mass selective detector. For HPLC/UV analysis an Agilent Technologies 1100 Series equipped with a G1379A degasser, 1200 Series Quaternary pump G1379A, G1367A autosampler, G1330B autosampler thermostat, G1316A thermostatted column compartment and a G1315B Diode Array Detector was used.

### Protein Expression

A codon optimized synthetic gene coding for *N*‐terminally HIS‐tagged NCBI accession # XP_955820.1 was ordered from GenScript custom cloned into the pETDUET1 vector with phosphopantetheinyltransferase from *E. coli* (NCBI accession # CAQ31055.1) in the upstream multiple cloning site. After sequencing, *E. coli* BL21 (DE3) Star or *E. coli* K12 MG1655 RARE was transfected with the plasmid pETDUET1:*Ec*PPTaseHT*Nc*CAR1 and colonies selected on LB/Amp. For protein expression, the autoinduction protocol as described by Studier^[34]^ was used. After 24 h at 20 °C, the cells were harvested by centrifugation and stored at −20 °C or used for biotransformations.

### Protein Purification

Thawed cells were disrupted by sonication and the protein purified by nickel affinity chromatography and concentrated *Nc*CAR protein preparations were stored at −80 °C in 50 mM MES buffer, pH 7.5, containing 10 mM MgCl_2_, 1 mM EDTA and 1 mM DTT. For determination of the kinetic parameters, the *Nc*CAR preparation obtained from Ni‐affinity purification was further purified by gel filtration on an ÄKTAPurifier 100. A HighLoad^TM^ 16/60 SuperdexTM 200 pg column was used (GE Healthcare) with a flow rate of 1 mL min^−1^ with 50 mM MES buffer, pH 7.5, containing 10 mM MgCl_2_, 1 mM EDTA, and 1 mM DTT as the mobile phase. Fractions of 1 mL were collected in a Frac 950 collector (GE Healthcare) and the protein containing fractions were pooled and stored at −80 °C.

### Spectrophotometric Assay

An NADPH depletion assay was used to screen for the substrate scope and to determine physicochemical characteristics of *Nc*CAR. Therefore, a number of carboxylic acids or carboxylate salts were dissolved in water, 0.1 M KOH or DMSO. The assay composition was as follows: the substrate (10 μL of 100 mM stock solution) was added to 160 μL of Tris‐HCl buffer (100 mM, pH 7.5, containing 10 mM MgCl_2_). Subsequently, 10 μL of NADPH (10 mM in water), 10 μL of ATP (20 mM in water) and 10 μL of CAR enzyme preparation from Ni‐affinity chromatography (0.2–0.7 mg mL^−1^) were added. The depletion of NADPH was followed on a Synergy Mx Platereader (BioTek) at 340 nm and 28 °C for 10 min. Appropriate blank reactions were carried out in parallel and each reaction was carried out at least in triplicate.

For the determination of the pH optimum, the standard assay as described above was used with cinnamic acid **1a** (100 mM in 0.1 M KOH) as the substrate and the following buffers: sodium citrate (pH 4.0, pH 4.5, pH 5.0 and pH 5.5), MES (pH 5.5 and pH 6.0), Tris‐HCl (pH 7.0 and pH 7.5) and glycine (pH 9.0, pH 9.5 and pH 10.0).

For the determination of kinetic parameters, the standard assay as described above was used with 160 μL of MES buffer (100 mM, containing 10 mM MgCl_2,_ pH 6.0) and CAR enzyme preparation after gel filtration (0.1–0.3 mg mL^−1^). Cinnamic acid **1a** dissolved in DMSO was used as a substrate. The final DMSO content was 5% v/v and **1a** concentrations were 0.00625, 0.0125, 0.025, 0.05, 0.1, 0.2, 0.4, 0.8, 1.6, 3.2, 6.4 and 12.8 mM, respectively. **2a** was dissolved in water in concentrations of 0.005, 0.05, 0.075, 0.125, 0.2, 0.25, 0.3, 0.5, 2.0, 5.0, 10 and 15 mM containing equimolar amounts of KOH, respectively. K_m_ and v_max_ were calculated on the basis of non‐linear regression by using Sigma Plot 11.0.

### Protein Melting Points Analysis

The nanoDSF grade standard glass capillaries were filled with *Nc*CAR in 50 mM MES buffer, pH 7.5, containing 10 mM MgCl_2_, 1 mM EDTA, and 1 mM DTT. A temperature gradient of 1 °C min^−1^ was applied and the intrinsic fluorescence analyzed at 350 nm in a Prometheus NT.48 nanoDSF device (NanoTemper Technologies).

### Chromatographic assays

The typical assay to determine directly the reaction products was carried out as follows: to 334 μL of 50 mM MES buffer, pH 7.5, containing 10 mM MgCl_2_, 1 mM EDTA, and 1 mM DTT, 16 μL of substrate solution (100 mM in 0.1 M KOH or DMSO) was added followed by 10 μL of *Nc*CAR preparation (approximately 0.02 mg mL^−1^), 20 μL of NADPH (80 mM in ddH_2_O) and 20 μL of ATP (80 mM in ddH_2_O). The reactions proceeded at 28 °C and 300 rpm in Eppendorf Thermomixers. The reactions were stopped by the addition of MeOH and vortexing. After centrifugation of precipitated protein, the supernatants were analyzed.

The analysis of *E*‐cinnamic acid (**1a**), *E*‐cinnamaldehyde (*E*‐3‐phenylprop‐2‐enal, **1b**) and cinnamyl alcohol (3‐phenyl‐2‐propen‐1‐ol, **1c**) was carried out with a Kinetex 2.6μ Biphenyl 100A HPLC column (Phenomenex) with a Phenylhexyl Security Guard ULTRA cartridge (Phenomenex). The mobile phases were ammonium acetate (5 mM) and 0.5% v/v acetic acid in water and ACN at a flow‐rate of 0.4 mL min^−1^. A stepwise gradient was used: 15–50% ACN (5 min) and 50–90% ACN (5.0–9.0 min). After 30 s, the column was re‐equilibrated to starting conditions. The compounds were detected at 254 nm (VWD) and negative scan mode (API‐ES) as well as single ion monitoring of the acid (M−1 147), the aldehyde (M+1 133) and the alcohol (M−1 133). For **1a**, **1b** and **1c**, calibration curves were determined at 254 nm and linear interpolation used for their quantification.

The analysis of piperonylic acid (1,3‐benzodioxole‐5‐carboxylic acid, **2a**), piperonal (heliotropin, 1,3‐benzodioxole‐5‐carboxaldehyde, **2b**) and piperonyl alcohol (1,3‐benzodioxole‐5‐methanol, **2c**) was carried out with a Kinetex 2.6μ Biphenyl 100A HPLC column (Phenomenex) with a Phenylhexyl Security Guard ULTRA cartridge (Phenomenex). The mobile phases were ammonium acetate (5 mM) and 0.5% v/v acetic acid in water and ACN at a flow‐rate of 0.26 mL min^−1^. A stepwise gradient was used: 25–55% ACN (5 min), 55–70% ACN (5.0–7.2 min) 70–90% ACN (7.2–7.5 min). After 90 s, the column was re‐equilibrated to starting conditions. The compounds were detected at 254 nm (DAD). For **2a**, **2b** and **2c**, calibration with authentic standards was determined at 254 nm and linear interpolation used for their quantification.

### Time‐Resolved ^31^P NMR

**Figure 6:**
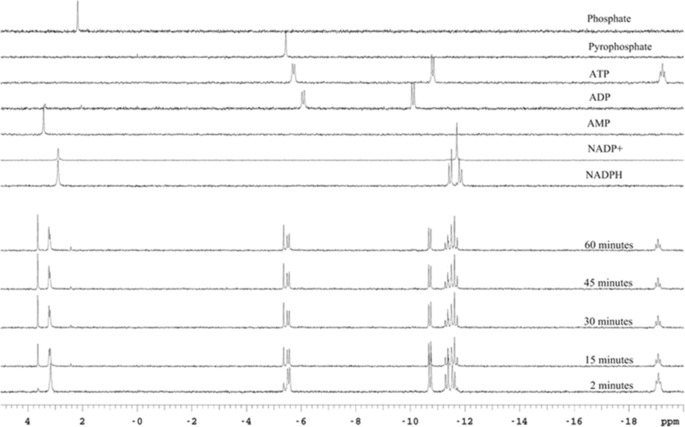
^31^P NMR reference spectra of all phosphorus‐containing species potentially involved in ATP and NADPH dependent *Nc*CAR mediated **1a** reduction and time resolved reaction.

^31^P NMR spectra were recorded at 202.354 MHz on an INOVA NMR spectrometer (Varian/Agilent) equipped with a direct detection multinuclear probe as described previously.^36^ NADPH, NADP^+^, AMP, ADP, ATP, K_2_HPO_4_, Na_4_P_2_O_7_ and MgCl_2_ were dissolved in TrisHCl buffer (100 mM, pH 7.5). Reference samples were composed of 335 μL of Tris‐HCl buffer (100 mM, pH 7.5), 50 μL of MgCl_2_ (200 mM), 25 μL MES buffer (100 mM, pH 7.5, 10 mM MgCl_2_, 1 mM EDTA, 1 mM DTT), 40 μL KOH (0.1 M in water), 50 μL D_2_O, and 50 μL of NADPH, NADP^+^, AMP, ADP, ATP, phosphate or pyrophosphate solution (100 mM), respectively. The biotransformation sample at time 0 was composed of 285 μL of Tris‐HCl buffer (100 mM, pH 7.5), 50 μL of MgCl_2_ (200 mM), 25 μL *Nc*CAR preparation (3.85 mg mL^−1^), 50 μL ATP (100 mM), 50 μL NADPH (100 mM), and 50 μL D_2_O. The reaction was started by addition of 40 μL of cinnamic acid **1a** (100 mM in 0.1 M KOH) (Figure 6).

### Resting Cell Biotransformations

Electrocompetent *E. coli* K‐12 MG1655 RARE cells were transfected with pETDUET1:*Ec*PPTase‐HT*Nc*CAR1 and the cells cultivated as described above. The initial biotransformation conditions (Figure 3, A) were 50 mM potassium phosphate buffer at pH 7.4 supplemented with 60 mM glucose, 70 mM citrate, 2 mM Mg^2+^ and 5 mM cinnamic acid (**1a**). 15 OD_600_ units (approximately 15 mg of cww mL^−1^) were used. The reaction was carried out in 24‐well multidish plates (Thermo Fisher Scientific) in a total volume of 1 mL. For the biotransformation of cinnamic acid (**1a**) to cinnamaldehyde (**1b**) different adjustments to the initial conditions were made. The concentration of glucose and the concentration of Mg^2+^ seem to have little influence on the conversion of **1a**. The citrate concentration was lowered to 10 mM because a lower **1b** yield and a higher amount of alcohol (**1c**) was detected in biotransformations with citrate concentrations ≥20 mM. Different amounts of cells (15–100 mg cww mL^−1^) were tested and 20 mg cww mL^−1^ was the optimal cell density. The addition of *n*‐hexane up to a 50% of the total volume showed the highest improvement on the yield of **1b**. The reaction with *n*‐hexane was carried out in 15‐mL Pyrex tubes and incubated at 28 °C in a tissue culture tube rotator for 3 h. The reaction with a total volume of 1 mL contained 50 mM potassium phosphate buffer at pH 7.4 supplemented with 60 mM glucose, 10 mM citrate, 2 mM Mg^2+^, 5 mM **1a** and 500 μL of *n*‐hexane. 20 OD_600_ units (about 20 mg of mg cww mL^−1^) were used per tube.

Typical experiments towards **2a** reduction were carried out in 100‐mL baffled flasks and contained 50 mM potassium phosphate buffer at pH 7.4 supplemented with 120 mM glucose, 20 mM citrate, 4 mM Mg^2+^ and 10–50 mM **2a** in a total volume of 10 mL. 500 OD_600_ units (about 50 mg cww mL^−1^) were used per flask. The flasks were agitated at 100 rpm and 28 °C in a Certomat BS‐1.

### Preparative Scale Biotransformations

Reactions were carried out in two baffled 2‐L flasks in a volume of 200 mL (0.99 g of **2a** per flask). The flasks were agitated at 100 rpm and 28 °C in a Certomat BS‐1. The reactions contained 50 mM potassium phosphate buffer pH 7.4, 120 mM glucose, 20 mM citrate, 4 mM Mg^2+^, 30 mM of **2a** and 50 g L^−1^ of fresh wet cells. Reduction of **2a** to **2b** was complete before 3 h according to HPLC analysis. Piperonal **2b** was extracted into *n*‐hexane directly from the biotransformation broth and crystallized as colourless needles in >98% purity; yield: 1.66 g (92%). ^1^H NMR (CDCl_3_): *δ*=9.83 (s, 1 H), 7.44 (d, 1 H, *J*=7.9 Hz), 7.36 (s, 1 H), 6.95 (d, 1 H, *J*=7.9 Hz), 6.10 (s, 2 H); ^13^C NMR (CDCl_3_): *δ*=190.3, 153.1, 148.7, 131.9, 128.7, 108.4, 106.9, 102.1.

## Supporting information

As a service to our authors and readers, this journal provides supporting information supplied by the authors. Such materials are peer reviewed and may be re‐organized for online delivery, but are not copy‐edited or typeset. Technical support issues arising from supporting information (other than missing files) should be addressed to the authors.

Supplementary

## References

[1] H. C. Brown , B. C. S. Rao , J. Am. Chem. Soc. 1958, 80, 5377–5380.

[2] S. Chandrasekhar , M. S. Kumar , B. Muralidhar , Tetrahedron Lett. 1998, 39, 909–910.

[3] J. Pritchard , G. A. Filonenko , R. van Putten , E. J. M. Hensen , E. A. Pidko , Chem. Soc. Rev. 2015, 44, 3808–3833. 25941799 10.1039/c5cs00038f

[4] K. Napora-Wijata , G. A. Strohmeier , M. Winkler , Biotechnol. J. 2014, 9, 822–843. 24737783 10.1002/biot.201400012

[5] A. He , T. Li , L. Daniels , I. Fotheringham , J. P. N. Rosazza , Appl. Environ. Microbiol. 2004, 70, 1874–1881. 15006821 10.1128/AEM.70.3.1874-1881.2004 PMC368342

[6] M. K. Akhtar , N. J. Turner , P. R. Jones , Proc. Natl. Acad. Sci. USA 2013, 110, 87–92. 23248280 10.1073/pnas.1216516110 PMC3538209

[7] M. Wang , H. Zhao , ACS Catal. 2014, 4, 1219–1225. 24804152 10.1021/cs500039v PMC3985451

[8] Y. Duan , P. Yao , X. Chen , X. Liu , R. Zhang , J. Feng , Q. Wu , D. Zhu , J. Mol. Catal. B: Enzym. 2015, 115, 1–7.

[9] Y. Duan , P. Yao , Y. Du , J. Feng , Q. Wu , D. Zhu , Beilstein J. Org. Chem. 2015, 11, 2245–2251. 26664647 10.3762/bjoc.11.243 PMC4661009

[10] M. Winkler , C. K. Winkler , Monatsh. Chem. Chem. Mon. 2016, 147, 575–578. 10.1007/s00706-016-1676-z PMC4785219 27069283

[11] M. Moura , D. Pertusi , S. Lenzini , N. Bhan , L. J. Broadbelt , K. E. J. Tyo , Biotechnol. Bioeng. 2016, 113, 944–952. 26479709 10.1002/bit.25860

[12] C. Li , Y. Matsuda , H. Gao , D. Hu , X. S. Yao , I. Abe , ChemBioChem 2016, 17, 904–907. 26972702 10.1002/cbic.201600087

[13] T. S. Raman , E. R. Shanmugasundaram , J. Bacteriol. 1962, 84, 1339–1340. 13990630 10.1128/jb.84.6.1339-1340.1962 PMC278069

[14] A. Enoki , Y. Yajima , M. H. Gold , Phytochemistry 1981, 20, 1543–1546.

[15] N. Kato , H. Konishi , M. Masuda , E.-H. Joung , M. Shimao , C. Sakazawa , J. Ferment. Bioeng. 1990, 69, 220–223.

[16] M. A. Palazzolo , M. L. Mascotti , E. S. Lewkowicz , M. Kurina-Sanz , J. Ind. Microbiol. Biotechnol. 2015, 42, 1581–1589. 26445878 10.1007/s10295-015-1696-4

[17] L. da S. Amaral , E. Rodrigues-Filho , J. Mol. Catal. B: Enzym. 2015, 113, 90–94.

[18] E. Brenna , F. Cannavale , M. Crotti , F. Parmeggiani , A. Romagnolo , F. Spina , G. C. Varese , J. Mol. Catal. B: Enzym. 2015, 116, 83–88.

[19] D. M. Bachman , B. Dragoon , S. John , Arch. Biochem. Biophys. 1960, 91, 326. 10.1016/0003-9861(60)90508-7 13685639

[20] G. G. Gross , M. H. Zenk , Eur. J. Biochem. 1969, 8, 413–419. 4389863 10.1111/j.1432-1033.1969.tb00543.x

[21] G. G. Gross , K. H. Bolkart , M. H. Zenk , Biochem. Biophys. Res. Commun. 1968, 32, 173–178. 4386171 10.1016/0006-291x(68)90365-3

[22] G. G. Gross , FEBS Lett. 1971, 17, 309–311. 11946054 10.1016/0014-5793(71)80172-2

[23] G. G. Gross , Eur. J. Biochem. 1972, 31, 585–592. 4405494 10.1111/j.1432-1033.1972.tb02569.x

[24] P. Venkitasubramanian , L. Daniels , J. P. N. Rosazza , J. Biol. Chem. 2007, 282, 478–485. 17102130 10.1074/jbc.M607980200

[25] R. Yamagishi , A. Yokomaku , F. Omoto , K. Misao , K. Takada , S. Yoshimatsu , A. Abe , M. Hayashi , Sleep Biol. Rhythms 2010, 8, 254–260.

[26] P. J. Frosch , J. D. Johansen , T. Menné , C. Pirker , S. C. Rastogi , K. E. Andersen , M. Bruze , A. Goossens , J. P. Lepoittevin , I. R. White , Contact Dermatitis 2002, 47, 279–287. 12534532 10.1034/j.1600-0536.2002.470204.x

[27] R. Japón-Luján , M. D. Luque de Castro , J. Chromatogr. A 2006, 1136, 185–191. 17045596 10.1016/j.chroma.2006.09.081

[28] B. L. Bowles , V. K. Juneja , J. Food Saf. 1998, 18, 101–112.

[29] W. H. Redd , S. L. Manne , B. Peters , P. B. Jacobsen , H. Schmidt , J. Magn. Reson. Imaging 1994, 4, 623–626. 7949692 10.1002/jmri.1880040419

[30] F. A. Beckford , J. Thessing , M. Shaloski , P. C. Mbarushimana , A. Brock , J. Didion , J. Woods , A. Gonzalez-Sarrías , N. P. Seeram , J. Mol. Struct. 2011, 992, 39–47. 21552381 10.1016/j.molstruc.2011.02.029 PMC3086094

[31] Z. Shi , Y. Li , Y. Kang , G. Hu , C. Huang-Fu , J. Deng , B. Liu , Acta Pharmacol. Sin. 2012, 33, 271–278. 22301863 10.1038/aps.2011.158 PMC4010342

[32] J. L. R. De Melos , E. C. Torres-Santos , V. D. S. Faiões , C. De Nigris Del Cistia , C. M. R. Sant'Anna , C. E. Rodrigues-Santos , A. Echevarria , Eur. J. Med. Chem. 2015, 103, 409–417. 26375353 10.1016/j.ejmech.2015.09.009

[33] D. E. Nichols , D. H. Lloyd , A. J. Hoffman , M. B. Nichols , G. K. Yim , J Med Chem 1982, 25, 530–535. 7086839 10.1021/jm00347a010

[34] F. W. Studier , Protein Expr. Purif. 2005, 41, 207–234. 15915565 10.1016/j.pep.2005.01.016

[35] T. Li , J. P. Rosazza , J. Biol. Chem. 1998, 273, 34230–34233. 9852085 10.1074/jbc.273.51.34230

[36] M. Winkler , K. Dokulil , H. Weber , T. Pavkov-Keller , B. Wilding , ChemBioChem 2015, 16, 2373–2378. 26391327 10.1002/cbic.201500335

[37] G. G. Gross , FEBS Lett. 1969, 5, 177–179. 11947270 10.1016/0014-5793(69)80325-x

[38] K. Napora-Wijata , K. Robins , A. Osorio-Lozada , M. Winkler , ChemCatChem 2014, 6, 1089–1095.

[39] A. M. Kunjapur , K. L. J. Prather , Appl. Environ. Microbiol. 2015, 81, 1892–1901. 25576610 10.1128/AEM.03319-14 PMC4345389

[40] J. N. Andexer , M. Richter , ChemBioChem 2015, 16, 380–386. 25619338 10.1002/cbic.201402550

[41] E. Cabiscol , J. Tamarit , J. Ros , Int. Microbiol. 2000, 3, 3–8. 10963327

[42] A. M. Kunjapur , Y. Tarasova , K. L. J. Prather , J. Am. Chem. Soc. 2014, 136, 11644–11654. 25076127 10.1021/ja506664a

[43] P. Venkitasubramanian , L. Daniels , S. Das , A. S. Lamm , J. P. N. Rosazza , Enzyme Microb. Technol. 2008, 42, 130–137. 22578862 10.1016/j.enzmictec.2007.08.009

