# Supplementary material for: Selective Enzymatic Transformation to Aldehydes in vivo by Fungal Carboxylate Reductase from Neurospora crassa
Source: Adv Synth Catal. 2016 Oct 4;358(21):3414–21. doi: 10.1002/adsc.201600914 (PMC5129534; doi:10.1002/adsc.201600914)
Supplement: Supplementary file 1 — Supplementary [file ADSC-358-3414-s001.pdf]

Advanced Synthesis & Catalysis

Supporting Information

Selective enzymatic transformation to aldehydes *in vivo* by fungal carboxylate reductase from *Neurospora crassa*

Daniel Schwendenwein, Giuseppe Fiume, Hansjörg Weber, Florian Rudroff, Margit Winkler*

SUPPLEMENTARY INFORMATION

Table of contents

1. Vector map pETDuet1-EcPPTaseHTNcCAR 2
2. Michaelis Menten curves 3
3. Reaction monitoring by HPLC 4
4. Reaction monitoring and product confirmation by NMR 6

1. Vector map pETDuet1-*Ec*PPTaseHT*Nc*CAR

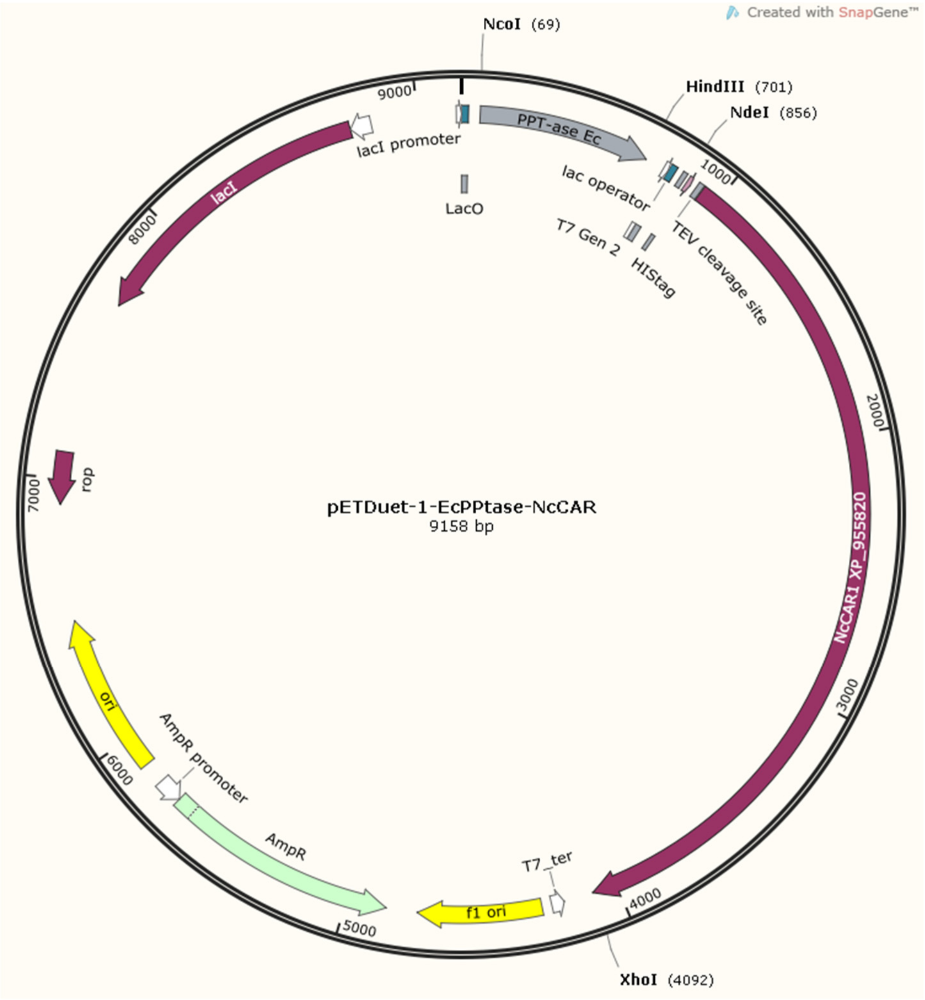

Figure S1. Illustration of the expression vector pETDuet1-*Ec*PPTaseHT*Nc*CAR. The plasmid encodes one T7 promoter in front of both expression cassettes, the T7 terminator behind the second expression cassette, an ampicillin resistance gene, the lacI gene for the two lac operators in front of every gene, a his tag at the 5' end of the *Nc*CAR and a pBR322-derived high copy origin of replication. The copy number is downregulated by the product of the rop gene and reaches a medium copy number. The two genes encoding *Ec*PPTase and *Nc*CAR were cloned in the two consecutive multiple cloning sites with *Nco*I and *Hind*III for *Ec*PPTase and *Nde*I and *Xho*I for *Nc*CAR.

2. Determination of kinetic parameters

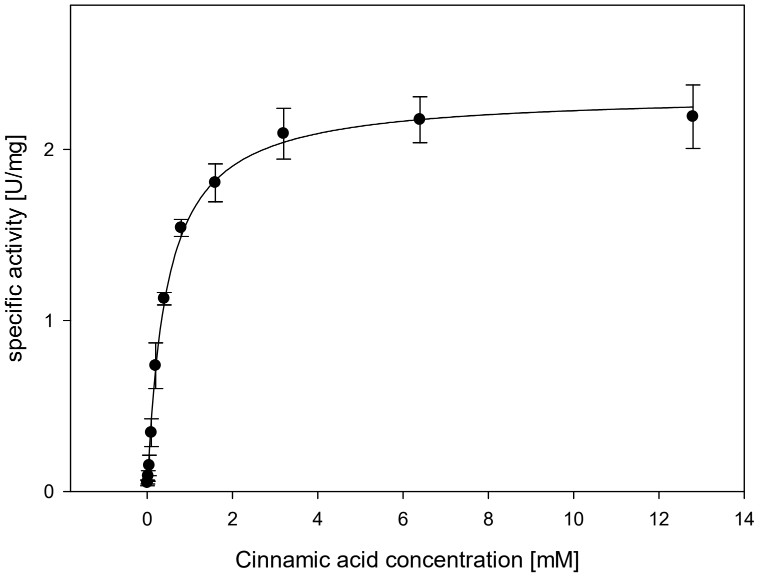

Figure S2 Saturation curve of the reduction of cinnamic acid **1a** dissolved in DMSO; K_m = 0.445±0.050 [mM], $v_{max,spec}$ = 2.33±0.06 [µmol min^{-1} mg^{-1}].

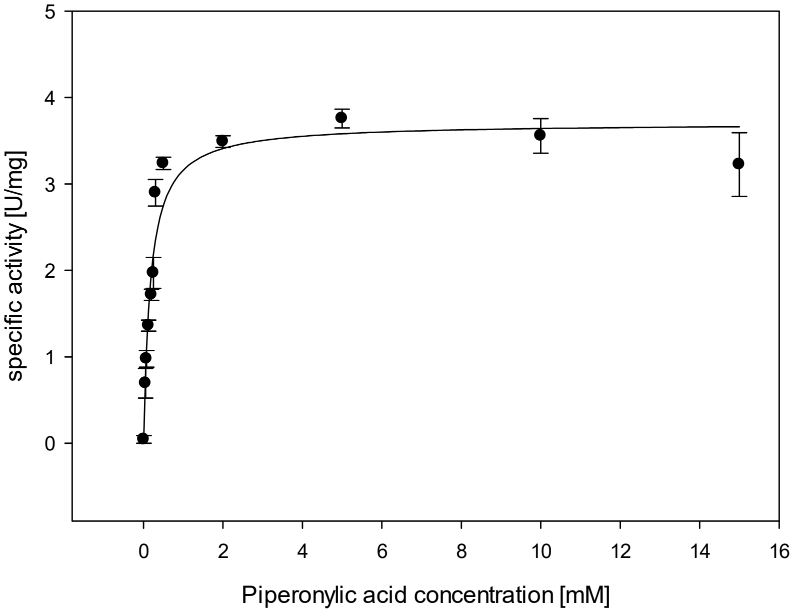

Figure S3 Saturation curve of the reduction of potassium piperonylate **2a** dissolved in water; K_m = 0.173±0.022 [mM], $v_{max,spec}$ = 3.70±0.12 [µmol min^{-1} mg^{-1}].

3. Reaction monitoring by HPLC

a. Determination of Cinnamic acid 1a reduction by HPLC/UV

The analysis of **1a**, **1b** and **1c** was carried out with a Kinetex 2.6µ Biphenyl 100A HPLC column (Phenomenex) with a Phenylhexyl Security Guard ULTRA cartridge (Phenomenex). The mobile phases were ammonium acetate (5 mM) and 0.5% v/v acetic acid in water and ACN at a flow-rate of 0.26 mL min^{-1}. A stepwise gradient was used: 25–55% ACN (5 min), 55–70% ACN (5.0–7.2 min) 70–90% ACN (7.2–7.5 min). After 90 s, the column was re-equilibrated to starting conditions. The compounds were detected at 254 nm (DAD). For **1a**, **1b** and **1c**, calibration with authentic standards was done at 254 nm and linear interpolation used for their quantification.

b. Effect of pyrophosphatase addition on cinnamic acid conversion

Inhibition by pyrophosphate was studied by comparison of the *Nc*CAR mediated conversion of cinnamic acid **1a** with or without the addition of commercial inorganic pyrophosphatase from baker's yeast (Sigma). *Nc*CAR preparation after gel-filtration with a concentration of 0.35 mg mL^{-1} was used for these experiments (0.0175 mg mL^{-1} final concentration). The standard spectrophotometric assay was used and the time course of the reaction was monitored by analysis of duplicate samples that were taken after 30, 60, 90, 120 and 240 minutes. In parallel, the reactions were carried out in the presence of pyrophosphatase with a final concentration of 0.0175 mg mL^{-1}. The results are depicted in Figure S4.

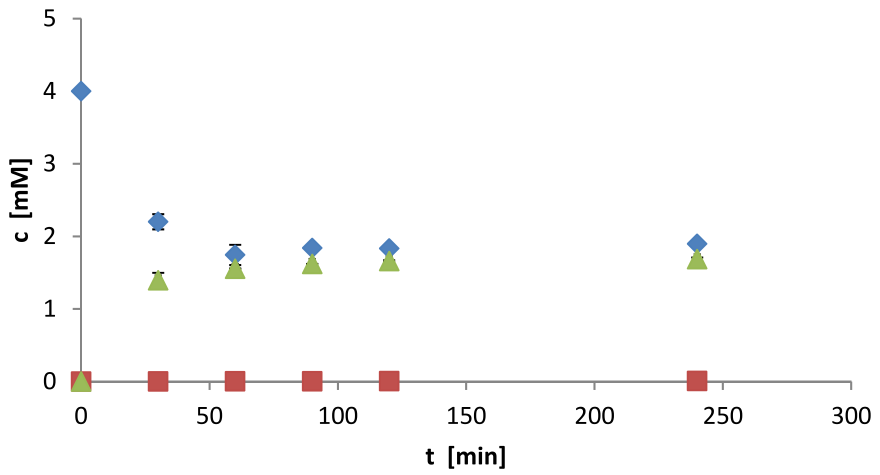

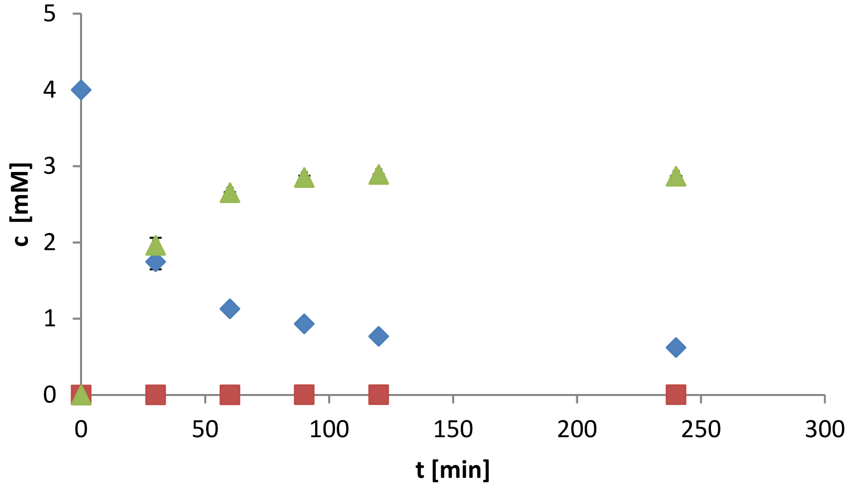

Figure S4 Time course of cinnamic acid **1a** reduction using *Nc*CAR *in vitro*. Blue diamonds represent **1a** concentrations, green triangles represent aldehyde **1b** concentrations and red squares represent alcohol **1c** concentrations. Top: without addition of inorganic pyrophosphatase; Bottom: with addition of inorganic pyrophosphatase.

c. Determination of Vanillic acid reduction by HPLC/MS

The analysis of vanillic acid (4-Hydroxy-3-methoxybenzoic acid), vanillin (4-hydroxy-3-methoxybenzaldehyde) and vanillyl alcohol (4-Hydroxy-3-methoxybenzyl alcohol) was carried out with a Kinetex 2.6µ Biphenyl 100A HPLC column (Phenomenex) with a Phenylhexyl Security Guard ULTRA cartridge (Phenomenex). The mobile phases were ammonium acetate (5 mM) and 0.5% v/v acetic acid in water and ACN at a flow-rate of 0.38 mL min^{-1}. A stepwise gradient was used: 0–20% ACN (5 min), 20–70% ACN (5.0–7.0 min) and 70–90% ACN (7.0–9.0 min). After 30 s, the column was re-equilibrated with aqueous phase. The compounds were detected at 254 nm (VWD) and negative scan mode (API-ES) as well as single ion monitoring of the acid (M-1 167), the aldehyde (M+1 153) and the alcohol (M-1 153).

4. Reaction monitoring and product confirmation by NMR

a. ^1H and ^{13}C NMR

When **1a** reduction did not proceed any further as judged by ^{31}P NMR, an ^1H NMR was measured to assess the progress of cinnamic acid **1a** reduction.

Figure **S5** shows both cinnamic acid **1a** and the respective aldehyde **1b** in approximately 2:1 ratio.

Piperonal was analyzed by ^1H and ^{13}C NMR, respectively (Figure S6 and Figure S7).

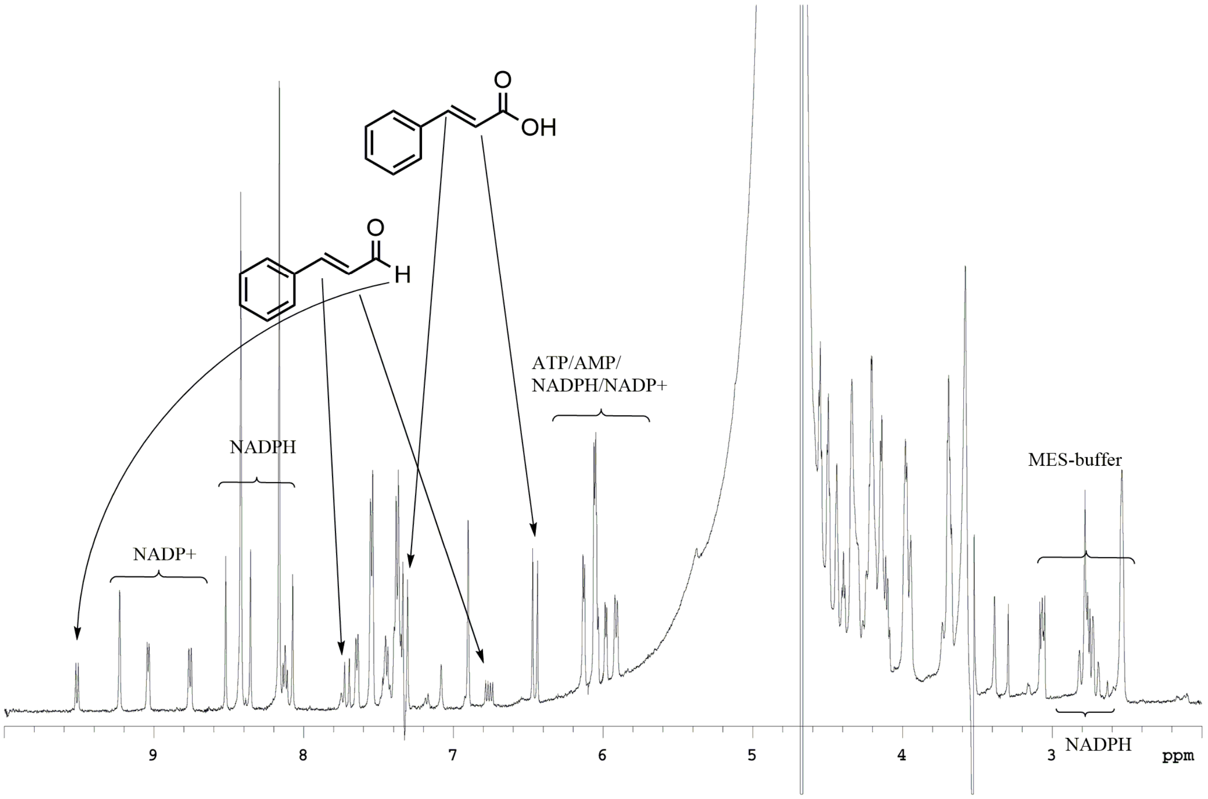

Figure S5 ^1H NMR of *Nc*CAR mediated reduction of cinnamic acid 1a after 4 h reaction time.

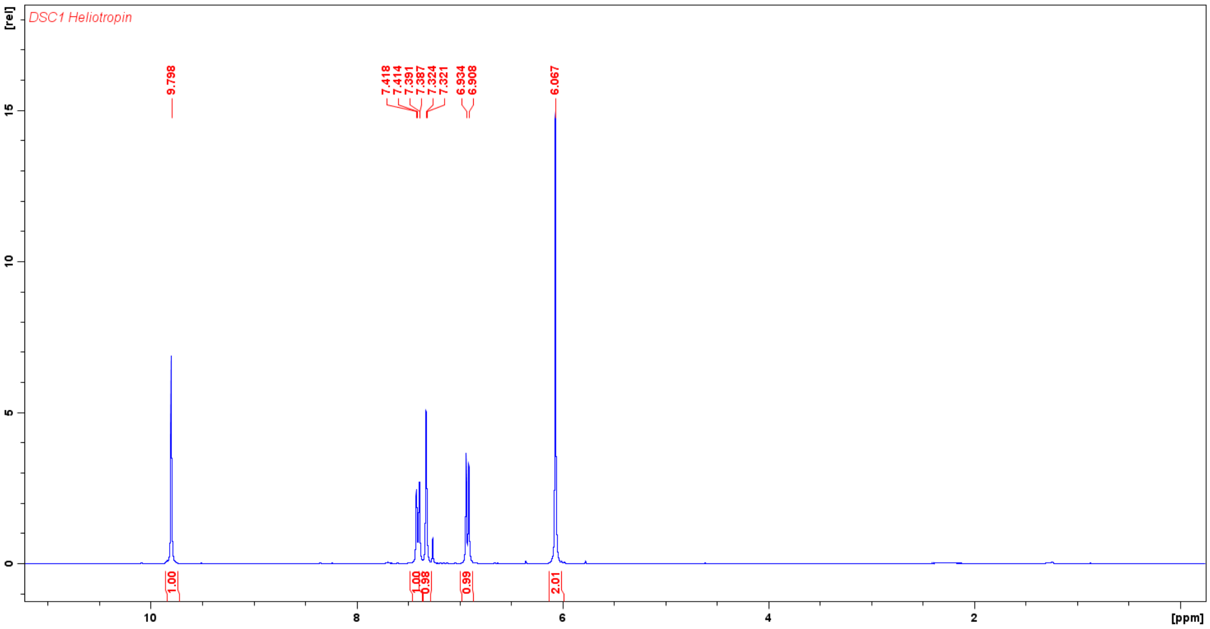

Figure S6. ^{1}H NMR of piperonal **2b** after *Nc*CAR mediated reduction from piperonylic acid **2a**, extraction into *n*-hexane and crystallization

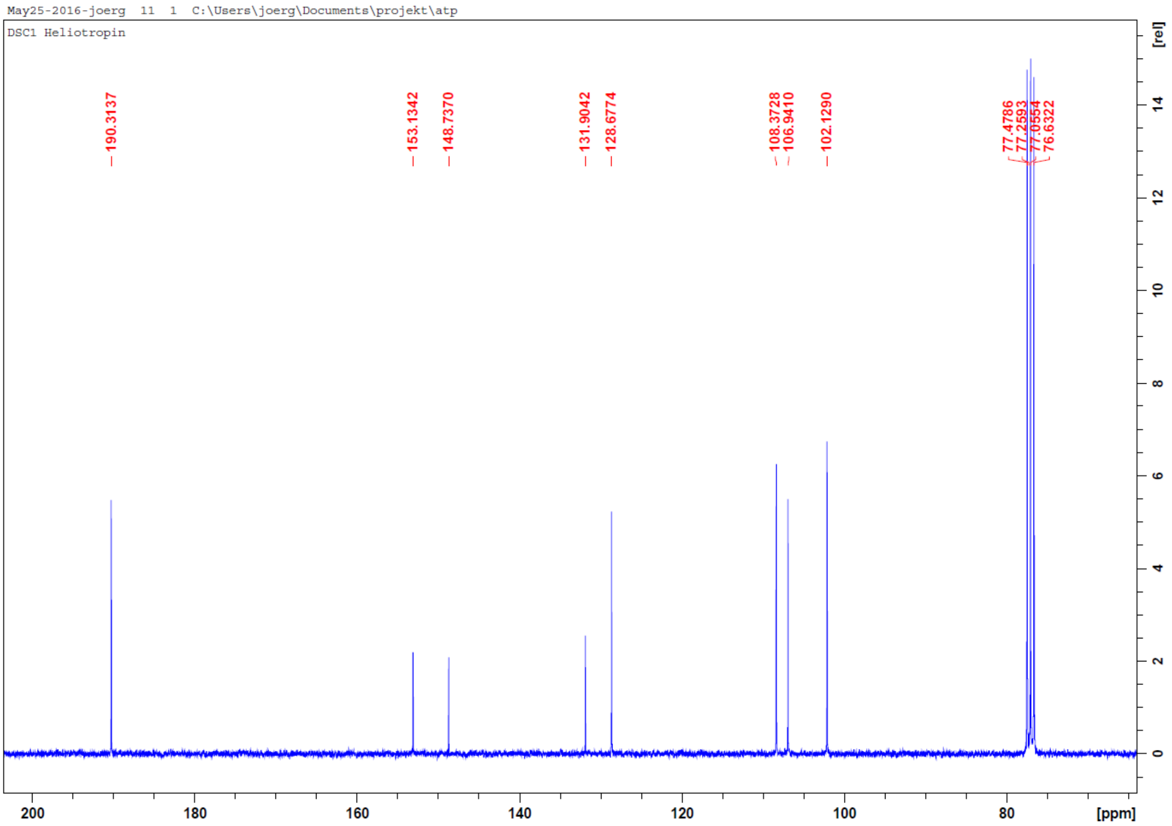

Figure S7 ^{13}C NMR of piperonal **2b** after *Nc*CAR mediated reduction from piperonylic acid **2a**, extraction into *n*-hexane and crystallization.
